# A Modular Sensorized Mat for Monitoring Infant Posture

**DOI:** 10.3390/s140100510

**Published:** 2013-12-31

**Authors:** Marco Donati, Francesca Cecchi, Filippo Bonaccorso, Marco Branciforte, Paolo Dario, Nicola Vitiello

**Affiliations:** 1 The BioRobotics Institute, Scuola Superiore Sant'Anna, viale Rinaldo Piaggio 34, Pontedera (PI) 56025, Italy; E-Mails: f.cecchi@sssup.it (F.C.); filippo.bonaccorso@sssup.it (F.B.); p.dario@sssup.it (P.D.); n.vitiello@sssup.it (N.V.); 2 ST Microelectronics, Stradale Primosole 50, Catania (CT) 95121, Italy; E-Mail: marco.branciforte@st.com

**Keywords:** pressure-sensitive mat, pressure-sensitive technology, optoelectronics, pressure maps, infant posture

## Abstract

We present a novel sensorized mat for monitoring infant's posture through the measure of pressure maps. The pressure-sensitive mat is based on an optoelectronic technology developed in the last few years at Scuola Superiore Sant'Anna: a soft silicone skin cover, which constitutes the mat, participates in the transduction principle and provides the mat with compliance. The device has a modular structure (with a minimum of one and a maximum of six sub-modules, and a total surface area of about 1 m^2^) that enables dimensional adaptation of the pressure-sensitive area to different specific applications. The system consists of on-board electronics for data collection, pre-elaboration, and transmission to a remote computing unit for analysis and posture classification. In this work we present a complete description of the sensing apparatus along with its experimental characterization and validation with five healthy infants.

## 1. Introduction

The concept of early intervention for the treatment of children and infants with developmental disabilities was introduced in clinical management some decades ago and its importance has increased considerably in the last three decades, thanks to new suggested methods of intervention and greater systematic use in clinical practice [1–6]. Early intervention offers the possibility of a positive influence on child development in the first months of life, thanks to early experience-dependent neuroplasticity [7]. For this reason, the importance of quickly starting intervention programs is generally emphasised.

Currently, the evaluation of the development of spontaneous motor activity in infants who present disabilities is carried out by specialized clinicians through visual analysis of rehabilitative tasks using functional-assessment scales. The quantitative analysis of the exercise is subject to the experience of the therapist and, therefore, not always easy. To introduce new parameters of infant's motor activity and coordination suitable for objective evaluation of patients, in the past 15 years several approaches were explored, mostly based on motion capture techniques. However, these approaches revealed a high level of intrusiveness since they required the placement of markers on the infant's body for motion tracking [8–11].

From an analysis of the state of the art [12–15], we concluded that a potentially more ecological solution to assess the rehabilitation path of infants is to monitor the changes in the infant's posture through the measurement of the pressure distribution (namely pressure map) from the interaction between the infant and the mat where he or she lays down during the therapy. This scientific objective can be framed in the wider research field of the measurement of interaction pressure in human-environment and human-robot interactions, such as the foot sole pressure map during gait, the interaction pressure between a hand and a handle, and the interaction pressure between a limb segment and a powered orthosis [16–18]. Although the interest in solutions for the measurement of the interaction pressure on wide surfaces has increased over recent years—in particular for applications in the domain of rehabilitation robotics [19]—recording pressure maps is a complex engineering task. Finding the best pressure sensor and technique is not easy, and several aspects have to be taken into account, such as the range of pressure of interest, the geometry of the contact area, and the accuracy and spatial resolution requirements. These considerations often prevent researchers from using off-the-shelf solutions and require the development of customized solutions for particular problems [20].

Thanks to their reliability and accuracy, force platforms and load cells are widely used in clinical fields and rehabilitation to measure interaction pressure [21,22]. However, they have several limitations such as size, weight, poor portability and/or, above all, the absence of information on the pressure distribution [20].

To overcome limitations introduced by force sensors and platforms, and thus collect more information on the distribution of pressure on a wide interaction area, pressure-sensitive technologies based on array of force/pressure sensors have been introduced in the literature [23,24]. We call these implementations pressure-sensitive mats.

There are different technologies available for the development of pressure-sensitive mats: in the state of the art we can find solutions for both industrial and clinical uses [12,25–29]. Among many variants, piezoresistive and capacitive solutions are the most common ones, given their high flexibility and cost effectiveness.

A piezoresistive polymeric film shows a decrease in electrical resistance when a normal force is applied. A technology based on this sensor is the Tekscan^®^ (Tekscan Inc., South Boston, MA, USA). Tekscan^®^'s sensorized mats are made of two thin flexible polymer sheets with embedded conductive lines, and contain thousands of sensing elements. They can measure pressure maps of non-uniform and rigid surfaces [30], and are commonly used in clinical trials to investigate postural control [31,32]. Nevertheless their sensors present several drawbacks: (i) drift of the output signal when the load is applied for long time [33–35]; (ii) output signal can change under loading conditions different from the one of the calibration phase [36,37]; (iii) and lack of a standard procedure for the calibration [38]. A similar sensing technology, based on force sensing resistors (FSR), was used by Harada *et al.* to develop a sensorized bed for adult [39] and infant [40] posture recognition, based on pressure distribution maps. The system is able to recognize three different postures (*i.e.*, supine, right lateral and left lateral) in the adult version, and the subject's status (*i.e.*, quiet, crying, moving) and his/her posture (*i.e.*, prone, supine), in the one for infants. Other solutions available in the state of the art have been conceived for monitoring the seated posture [41], gait analysis [42] or postural control [43]. These devices showed some limitations such as high non-linearity, sensitivity to temperature and humidity, and sensitivity of the input-output behaviour to the number of loading-unloading cycles and—more generally—to the loading history [44].

Capacitive sensors are generally composed of three layers: two conductive plates with a middle sensing layer made of non-conductive elastomer exhibiting high values of dielectric constant. In the literature, there are several capacitive mats developed to address different specific applications: classification of sitting posture on a chair [45], in-home sleep and movement monitoring [23], medical seating, tire design, and mattress retail industry [24]. Despite higher performance in terms of reliability, accuracy and robustness to calibration procedures, capacitive mats require high-cost and sophisticated electronics to sense small capacitive changes (on the order of 10^−12^ F) [45].

In this paper we present a novel modular pressure-sensitive mat for monitoring infant posture based on a pressure-sensitive optoelectronic transduction principle, developed at Scuola Superiore Sant'Anna in the last few years [17,18,20]. In particular, with reference to paper [20]—which addresses a review of the technology—we built the sensorized mat upon the sensing element of the second generation of pressure-sensitive pads (PSP), namely PSP2.1 (see Section 2.1). We opted for the use of this technology because it offers three main advantages compared to other state-of-the-art solutions. First, the output signal does not drift over long recording sessions. Second, the adopted technology does not require calibration at every experimental trial, but rather calibration is executed only once, after mat fabrication is completed. Third, the output signal of each sensing element does not require any amplification electronics. Furthermore, the adopted technology was also chosen because the pressure range of the PSP2.1 was comparable to the one required for the sensorized mat [20]. However, differently from the PSP2.1, the sensorized mat has: (i) a much higher number of sensors that required the implementation of a multiplexer; (ii) a rigid PCB; and (iii) a modular architecture relying on a data transmission routed through a wired (*i.e.*, USB) connection.

The pressure-sensitive mat was designed by addressing the following main design requirements. First, we wanted to monitor the posture changes of 4-to-8 month-old infants. Second, similar to the sensor density (0.25–1 sensor/cm^2^) of other existing devices [14,15,31,40], the required spatial resolution was about 1 cm^2^. Third, the sensing area should be modifiable and cover a surface ranging from about 0.18 m^2^ (0.43 × 0.43 m) to about 0.8 m^2^ (0.84 × 0.96 m) (both are typical values of the surface covered by the mat used by therapists to carry out the rehabilitation sessions [14,15]). The latter requirement was mostly addressed by endowing the system with a certain degree of modularity. Finally, in order to accomplish the goal of monitoring the movement of the infant while being in a certain posture, the sensorized mat has to be capable of transmitting consistent information about the spatial localization of the pressure spots from the interaction between the infant and the mat.

Finally, in this paper, along with the description of the system architecture, we carried out an experimental validation of the device with five healthy infants in order to assess the capability of the device to record pressure maps collecting consistent information about the infant's posture. On the other hand, it was out of the scope of this work to develop automatic algorithms, such as the algorithms presented in [40], which extract indexes from the collected maps for the assessment of the infants' motion.

This paper is structured as follows: Section 2 describes the pressure-sensitive mat architecture and main components. Section 3 reports on the results from the experimental characterization, from both bench-tests and the validation with five full-term healthy infants. Section 4 reports a discussion about the validation tests. Finally, Section 5 draws the conclusions and proposes some perspectives on future uses and further development of the system.

## 2. Architecture and Components of the Pressure-Sensitive Mat

**Figure 1.**
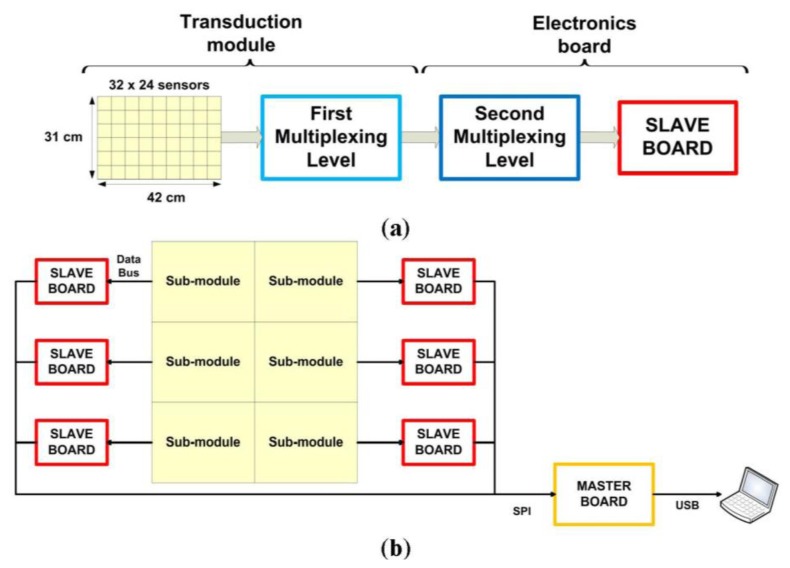
(**a**) Overview of the sub-module's architecture; (**b**) overview of the overall architecture of the sensorized mat.

The pressure-sensitive mat is composed of two main parts: the transduction module, *i.e.*, the printed circuit board implementing the array of sensitive elements, and the set of electronic boards for signal acquisition and conditioning, the latter being connected to a remote PC for data display and logging. Figure 1 shows an overview of the overall architecture, which is described in more details in the next paragraphs.

### 2.1. Transduction Module of the Pressure-Sensitive Mat

The transduction module is composed of a variable number of sub-modules (from a minimum of one to a maximum of six). Each sub-module integrates—on the top face—a pressure-sensitive array of 32 × 24 sensors (total amount of 768), for an overall sensing area of 31 × 42 cm^2^, and—on the bottom face—a first layer of multiplexers to pass from 768 to 48 analog input signals to the electronics board (see Figure 1a). The sensitive element is based on the last generation of the pressure-sensitive technology, namely PSP2.1, which is fully described in the recent work of Donati *et al.* [20]. A brief description is hereafter recapped for sake of clarity.

**Figure 2.**
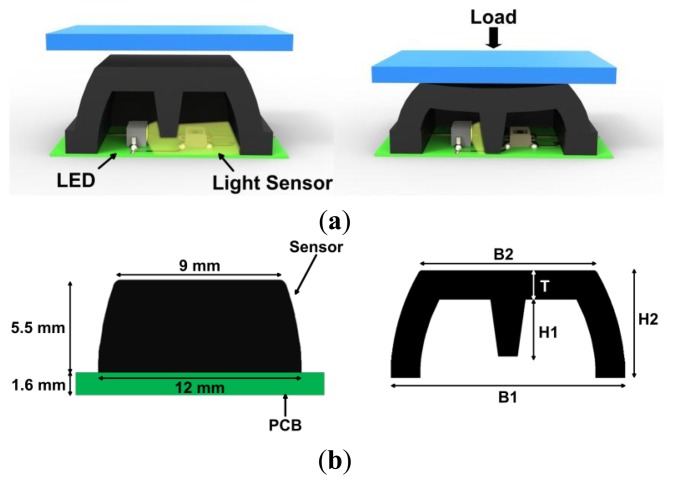
(**a**) Overview of the sensitive element and its functioning principle; (**b**) dimension and cross section of the silicone cover.

The pressure-sensitive array is composed of two main parts: (i) a silicone layer divided into independent cells; and (ii) a 1.6-mm-thick printed circuit board (PCB) which houses the optoelectronic components. Each independent silicone cell contains a pressure-sensitive element. The silicone cell assumes the shape of a pyramidal frustum with a square basis and an internal central curtain, which covers a couple of light emitter-receiver diodes. The light emitter is a high-luminosity green LED (Opto Semiconductor OSRAM, Munich, Germany [46]). The light receiver is an ambient-light photodiode (Avago Technologies Ltd., San Jose, CA, USA [47]). The silicone layer is opaque and plays an active role in the two-phase transduction mechanism of the pressure/force signal. First, when a force/pressure is progressively applied on the top surface of the frustum, the silicone bulk deforms itself (force-to-deformation conversion). Second, the curtain gradually closes the light pathway between the emitter and the receiver, and thus the output voltage changes (Figure 2a), namely deformation-to-output voltage conversion.

The dimension of the frustum base is 12 × 12 mm^2^ (the frustum base is surrounded by a frame of 1-mm of free space), while the top face is 9 × 9 mm^2^, and the height is 5.5 mm (Figure 2b). Given the size of the frustum base and the surrounding free space, the actual spatial resolution is 1.69 cm^2^. The shape of the cover is identified by five geometrical parameters: (i) the side of the lower base B1; (ii) the side of the upper face B2; (iii) the thickness T; (iv) the height of the curtain H1; (v) and the height of the frustum H2. As explained in [20], by changing the value of these parameters and/or the mechanical properties of the silicone, the sensitivity of the sensor to the applied load changes. A cross-section of the frustum is shown in Figure 2b.

The silicone layer was realized by using Dragon Skin 10 Medium (Shore 10 A, Smooth-On Inc., Easton, PA, USA), coloured by black pigment. The silicone was characterized by Axel Products Inc. (Ann Arbor, MI, USA), in order to define its basic elastomeric properties through the execution of four mechanical tests [48,49]: volumetric compression (using a cylindrical specimen that is constrained in a fixture and compressed), simple tension (using a long, thin specimen and a video or laser extensometer), pure shear (using a wide specimen and a video or laser extensometer), and equal biaxial stress (using a circular specimen stretched on radial direction and a video or laser extensometer). The tests were performed under slow cyclical loads to avoid the Mullin's effect (the structural properties of the silicone change during the first loading cycle) [50]. Data were used to define the nine-parameter Mooney-Rivlin solid model to create the 3-dimensional finite-element (3D FE) model in ANSYS 12 (Analysis Inc., Canonsburg, PA, USA).

**Figure 3.**
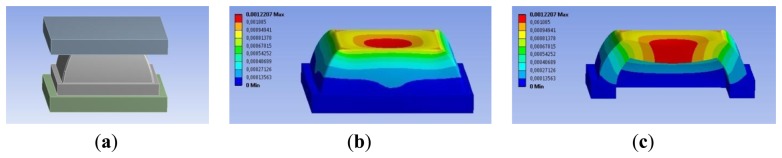
3-Dimensional finite-element model: (**a**) model of the sensitive element; (**b**) total deformation of the silicone structure; (**c**) detail of the sinking effect.

In order to identify the geometrical parameters shown in Figure 2b, we carried out iterative FE simulations. The desired sensing range was set to 3 N (*i.e.*, 0–30 kPa) and the maximum compression of the top face of the frustum to 1.1 mm. In each FE simulation a rigid flat indenter, parallel to the PCB, pushed on the top-face of the frustum with an increasing load (Figure 3a). The contact region between the indenter and the top-face was modelled as a rigid friction connection [51], and the region between the bottom-face and the PCB was modelled as fixed support. To simulate the load, a displacement of the indenter was imposed, and for each deformation state we evaluated the total stress state, the total force response, and the deformation state of the structure (see Figure 3b). The FE analysis also showed that the structure suffers from a sinking effect which—on the one hand—increases the sensitivity of the sensor for low values of applied force, and—on the other hand—reduces the sensing range. Indeed, smaller loads would cause the silicone cover to touch the PCB and, as a consequence, the sensor to saturate (see Figure 3c). For this reason, the choice of the geometrical parameters was defined by minimizing the sinking effect. The geometrical parameters that resulted from the iterative design process were: B1 = 12 mm, B2 = 9 mm, H1 = 2.9 mm, H2 = 5.5 mm, and T = 1.5 mm. The silicone cover was then obtained by casting liquid silicone in an acrylic mold. After the polymerization, the silicon cover was glued onto the PCB.

It is worth noting that the desired range of 30 kPa was derived from the following analysis. By considering the high inter- and intra-subject anthropometric variability of the target population, we assumed that body weight of the target population would be in the range of 4 to 10 kg (5 to 95 percentile). Data were extracted from the analysis of the growth chart for children published by the Centers for Disease Control and Prevention [52].

### 2.2. Electronic Boards

Every sub-module has a dedicated electronic board, slave board, for the acquisition and conditioning of pressure signals coming from the sensors. Slave boards are managed by a master board, which acquires signals and sends all data to a dedicated computer (see Figure 1b).

**Figure 4.**
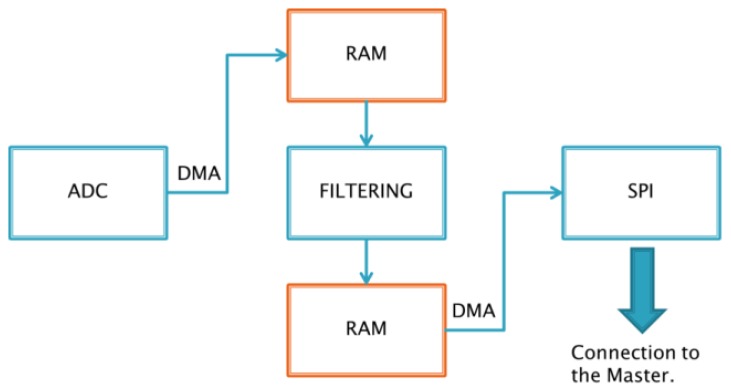
Overview of sub-module operations.

The core of the electronics, involved in acquiring and conditioning the 768 sensor signals of each sub-module, is an STM32F4 microcontroller unit [53], which is based on a 32-bit ARM Cortex™-M4 CPU (STMicroelectronics N.V., Genève, Switzerland). This can reach an operation frequency of 168 MHz allowing up to 210 MIPS. Among all its features this device includes DMA, FPU, and DSP, three analog/digital (A/D) converters with a maximum resolution of 12 bit and sampling rate of 2.4 MSPS, and three SPI interfaces able to communicate up to 37.5 Mbps. Figure 4 summarizes the operation performed by each slave sub-module.

**Figure 5.**
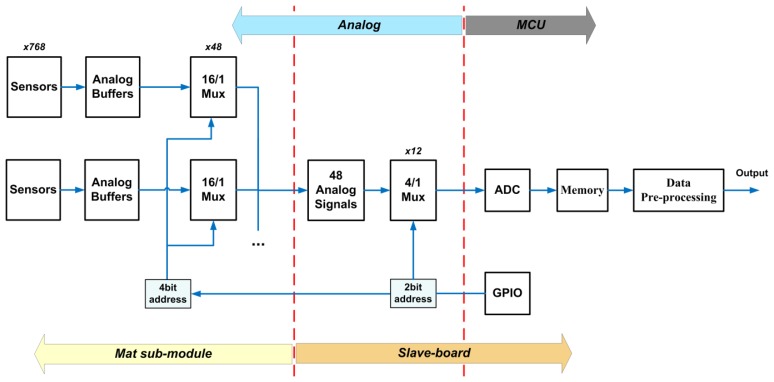
Overview of sub-module multiplexing architecture and data managing.

The output of the 768 sensors are arranged into 48 signals due to the multiplexing operations managed by the transduction module (first multiplexing level). These signals are routed through 6 connectors with 8 pins each. In order to reduce the number of signals to be managed by the microcontroller, the slave sub-module implements a second multiplexing level by 4-to-1 (see Figure 5). The design phase has taken into account several aspects such as multiplexer delay, possible A/D configurations and communication strategies. A trade-off has been obtained yielding a maximum sampling frequency, on all 768 sensors, for each sub-module, of 20 Hz.

A/D converters were configured to work with a 21-MHz clock and 12-bit resolution. The three converters work in parallel in order to acquire 12 signals at each converting stage, so that all sensors of each sub-module are acquired within 64 stages; before performing each A/D conversion the on-board MCU acts on the multiplexers, in order to select the right channels, and waits for the time needed to have a stable output on the multiplexers. Collected data are stored into 16-bit registers (all 768 signals need 1,536 Kbyte). Data are then sent to the master-board using an SPI connection which communicates at 10.5 Mbps.

Slave boards are managed by a master board which is also based on a STM32F4 microcontroller. The master-board addresses three main operations: (i) it triggers the acquisition phase of all connected slaves; (ii) it receives data from slave boards; and (iii) it communicates with the remote computer through a USB virtual com port (VCP). To perform all of these operations the master board implements a finite-state machine which is driven by the remote PC through a dedicated communication protocol built upon the VCP.

## 3. Experimental Characterization

**Figure 6.**
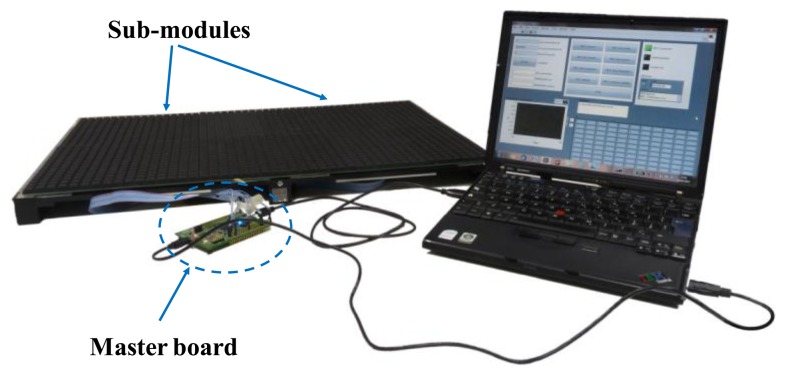
Sensorized mat housed onto a rigid plastic frame. The device is composed of: a master board, two sub-modules each endowed with its slave board (housed under the sensing area, within the plastic frame) and a laptop computer with a Labview interface for data logging.

In this Section we report the results from the experimental characterization of the developed pressure-sensitive mat. The device used for the tests was composed of a master board, two sub-modules and two slave boards: the overall sensing area was 42 × 62 cm^2^ (Figure 6). The experimental characterization consisted of two tests. First, we carried out a bench-test which aimed at constructing a numerical model of the force-to-voltage characterization curve of the sensing elements (as explained in the previous Section, the force-to-voltage conversion curve results from the two-stage transduction mechanism: force-to-deformation and deformation-to-voltage). Second, we evaluated the capability of the developed instrumentation to bring exhaustive information about the spatial localization of pressure spots for each of the three different postures (prone, supine and seated) of five healthy infants laid down on the mat.

### 3.1. Force-to-Voltage Characterization Curve of the Sensitive Element

In order to know the mechanical properties (*i.e.*, force *vs.* deformation behaviour) and the force-to-voltage calibration curve of the mat sensitive elements, it was necessary to carry out an experimental characterization [20]. For this aim we used a 3-axial platform (TAP) machine, developed at The BioRobotics Institute (Pontedera, Italy), which was equipped with a six-axis load-cell (ATI Nano-17 SI-25-0.25, ATI Industrial Automation, Apex, NC, USA), and a rigid flat indenter. The indenter was oriented parallel to the mat plane and a set of deformations were applied, mimicking the conditions we considered in the 3D finite-element model.

Since the silicon cover of one sub-module is comprised of six 8 × 16 smaller arrays molded separately, we expected that the force-to-output voltage behaviour could differ among the sensitive elements. As a consequence, we decided to carry out the experimental characterization on 12 out of 768 sensitive elements on one of the two sub-modules. In particular, we randomly selected two sensitive elements from each of the six 8 × 16 smaller arrays molded separately. This way, we could identify the calibration function (*i.e.*, the force-to-output voltage curve) by averaging the behaviour of a representative set of sensitive elements.

In order to identify the force-to-output voltage curve—for each selected sensitive element—we applied step-like loads resulting from deformations in the range of 0–1.3 mm, with a step size of 0.1 mm and a loading speed of 0.1 mm/s. For each step we recorded the applied force and the output voltage at the steady state.

The resulting data from each sensitive element were fitted by the sum of two exponential functions (*i.e.*, *F* = *A*_1_*e*^*c*_1_*V*^ + *F* = *A*_2_*e*^*c*_2_*V*^, where *F* is the applied force and *V* is the output voltage), which was found to be the best compromise in terms of complexity and goodness of fit (Matlab^®^ cftool). The same function was also used to fit the experimental data from all 12 sensitive elements grouped together.

**Figure 7.**
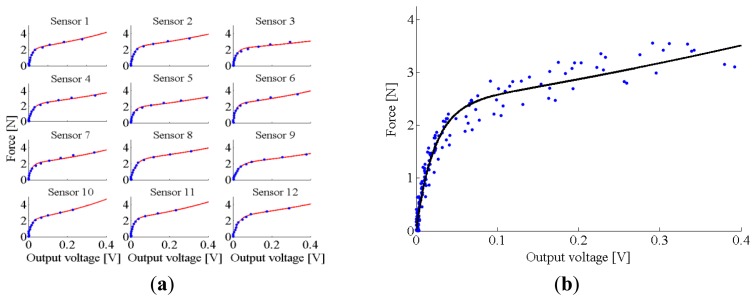
Force-to-output voltage characterization of the sensitive element: (**a**) experimental data (blue dots) and fitting model (solid red line) of the 12 selected sensors; (**b**) experimental data of the 12 selected sensors (blue dots) and averaged fitting model (solid black line).

In Figure 7a, we report the experimental data from all of the 12 sensitive elements along with the corresponding fitted model. In Figure 7b, we reported the model averaging the behaviour of the 12 selected sensitive elements. The coefficients of the fitted functions (along with parameters showing the goodness of the fit, *i.e.*, RMSE in N, R^2^ and RMSE expressed in % of the full-scale range) are summarized in Table 1.

**Figure 8.**
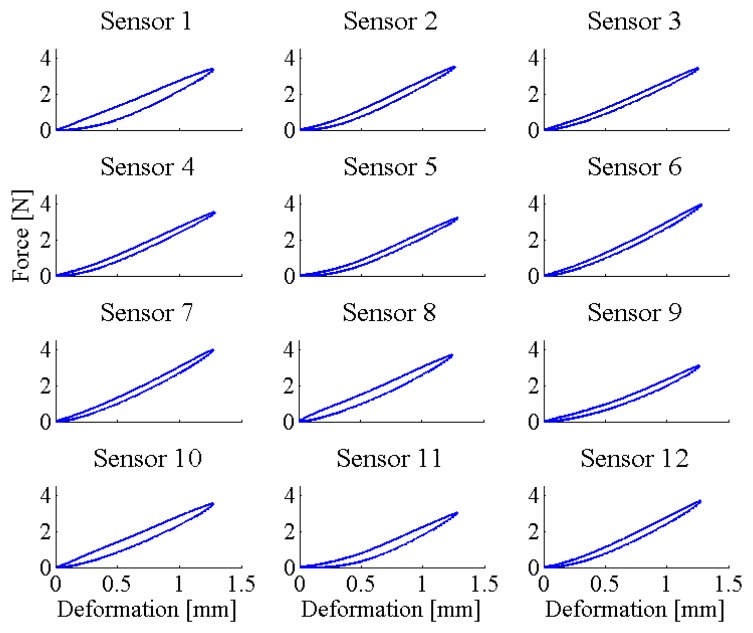
Deformation-to-force loading-unloading curves: the fitted curves are reported for each sensor.

In order to assess the variability of the mechanical properties (*i.e.*, force *vs.* deformation behaviour) among the mat sensitive elements, we assessed the mechanical hysteresis of the selected sensors under loading-unloading cycles. For this aim, we continuously loaded/unloaded in the range of 0–1.3 mm each sensor (loading speed equal to 0.1 mm/min) while measuring the applied force. All data were off-line low-pass filtered with a third-order Butterworth filter, with a cut-off frequency equal to 20 Hz (Matlab^®^ filtfilt function). All loading and unloading cycles were separately fitted with a third-order polynomial function—which was found to be the best compromise in terms of complexity and goodness of fit (results are shown in Figure 8). Hysteresis was then computed as the maximum difference between the fitted curve of the loading and unloading data. The resulting data on the hysteresis of each sensitive element are summarized in Table 2.

### 3.2. Experimental Validation with Healthy Infants

In order to assess the usability of the sensorized mat as a tool to monitor infants' posture, we carried out an experimental validation with healthy subjects. Five infants (age: 10–22 weeks, mean: 16 weeks; weight: 5.55–7.7 kg, mean: 6.5 kg, see Table 3) took part in the study upon parental consent; the experimental protocol was conducted according to the Declaration of Helsinki.

With the help of a parent, each infant was laid down onto the sensorized mat in three different postures, namely: prone, supine, and seated. For each subject and posture we recorded a steady-state 32 × 48 pressure map, by averaging the output voltage of each sensor over 0.5 s (raw data were recorded at 10 Hz). A custom Labview (National Instruments, Inc., Austin, TX, USA) routine was developed to receive and store data from the master board, and to offset the output voltages after the system power-on.

In order to emphasize pressure spots, collected pressure maps underwent the following two-step filtering procedure:

(1) the output voltage of the single sensitive element was set to zero if its value was lower than the noise threshold, *i.e.*, 0.01 V;
(2) the pressure map was then filtered by means of a median filter (Matlab^®^, medfilt2 function), to remove outliers, and a Gaussian filter (Matlab^®^, imfilter function), to smooth the pressure spots; output voltages were then converted into force.

**Figure 9.**
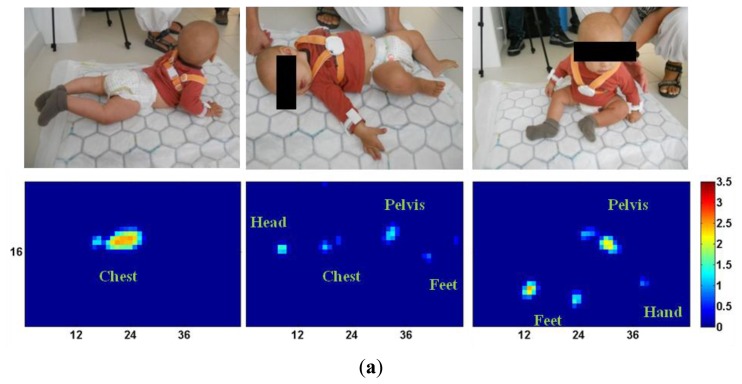

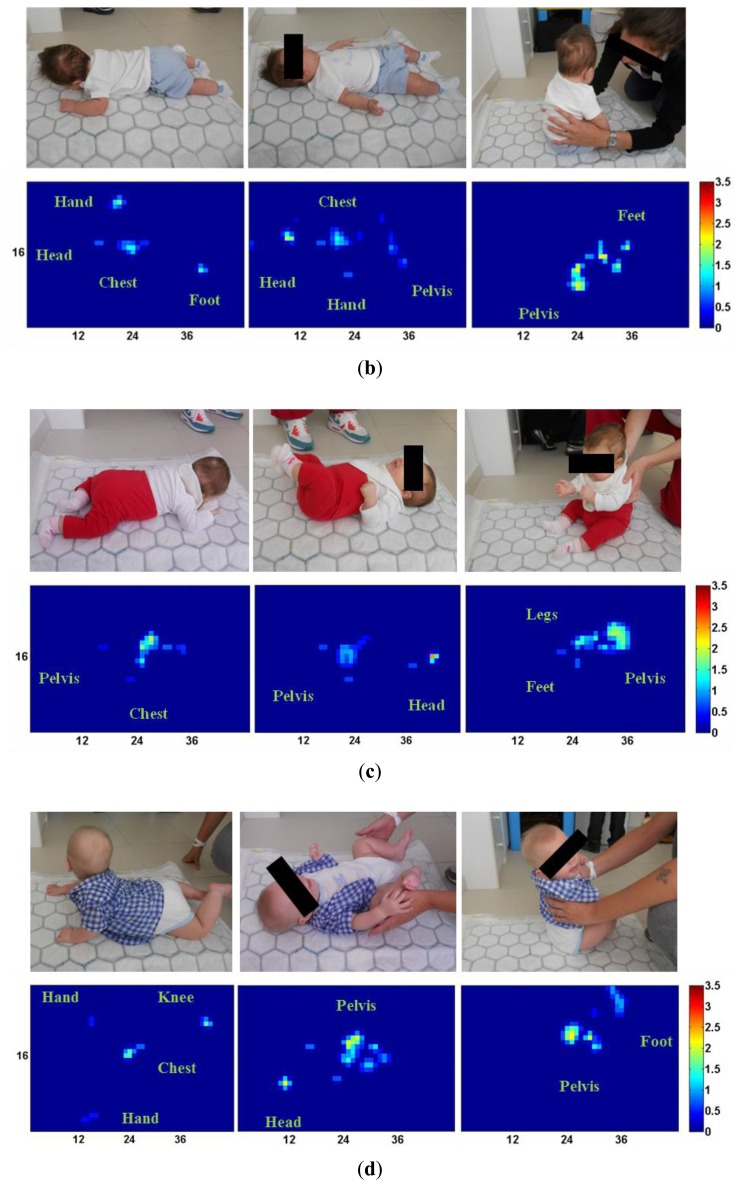

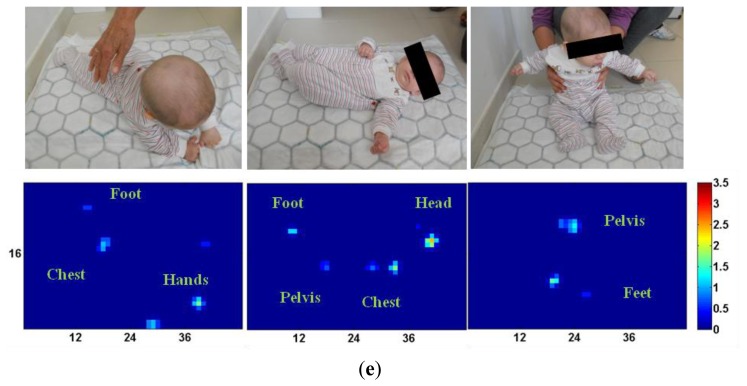
Infants' postures and pressure maps. (**a**) Subject #1; (**b**) Subject #2; (**c**) Subject #3; (**d**) Subject #4; (**e**) Subject #5. (Left column: *prone*; middle column: *supine*; right column: *seated*). Color scale is in [N].

In Figure 9 we reported all of the collected pressure maps: each pressure map is accompanied by a picture showing the actual infants' posture at steady state.

Furthermore, in order to estimate the actual error when “sensing” a distributed load applied onto the sensorized mat, we calculated and compared the total applied vertical force (*vRF*) with the body weight of the infants. The output voltage *v*_1_ of the i-th sensitive element de-offset and converted into a force *F_i_* is as follows:

(1) $$\{\begin{array}{c} F_{i}=0\;\text{N},\text{if}\;v_{i}<0.01\;\text{V}\\ F_{i}=2.352\cdot e^{0.999\cdot v_{i}}-2.326\cdot e^{-44.84\cdot v_{i}}\;\text{N},\text{if}\;v_{i}\geq 0.01\;\text{V} \end{array}$$

The *vRF* was calculated as $\text{vRF}=\sum_{i=1}^{i=M}F_{i}$, with *M* = 1,536 being the total number of sensitive elements. For each infant, we than averaged the *vRF* over a time interval of five s in which the infant had a quasi-steady posture. Collected data are reported in Table 4.

## 4. Discussion

In this paper we presented a novel sensorized mat for monitoring infants' posture through the measure of pressure maps. Along with the description of the system working principle and implementation, we carried out both bench tests and experiments with healthy infants to assess the overall system usability, namely the performance of the single sensitive element and the collection of pressure maps in a prototypical application scenario. Furthermore, the reliability of the pressure-sensitive technology in the estimation of the interaction pressure between the infant and the mat was assessed by comparing the total applied vertical force (*vRF*) with the body weight of the infants.

The experimental characterization of the sensitive element revealed that the sensitive range is about 0–35 kPa, with the output voltage increasing from 0 to about 0.4 V. The force-to-output voltage behaviour of each sensitive element is well modelled by a sum of two exponential functions: the maximum RMSE for one of the sensitive elements was about 0.2 N, corresponding to about 5% of the full-scale range. When looking at the numerical model resulting from the fit of all experimental data, the RMSE is slightly higher, *i.e.*, 0.22 N, corresponding to about 6% of the full scale range. Since the 12 characterized sensitive elements were randomly selected from the six 8 × 16 smaller arrays (two for each array) of the silicone cover (chosen from one of the two sub-modules), these results proved that the hand-made molding procedure—although it introduces variability in the force-to-output voltage behaviour—does not affect the usability of the system. Indeed, an error of about 6% of the full-scale range in the estimate of the force applied onto each sensitive element is not critical for recognizing spots in the pressure maps derived from infants' posture changes. For example, the expected steady-state pressure spot derived from the interaction of the infant's head, chest or pelvis with the mat is in the range of 10–25 kPa (we assume a weight of 10 N distributed over a surface ranging from 4 cm^2^ for the head to 10 cm^2^ for the pelvis), which is significantly higher than the maximum error introduced by the numerical model of all elements, namely 2 kPa.

It is worth noting that the variability of the sensitive elements is mostly the result of slightly different mechanical properties (*i.e.*, force *vs.* deformation behaviour) of the sensors. When looking at the results of the deformation-to-force characterization under cyclical loads, hysteresis ranges from 8% to 21% of the full-scale range. Despite this variability, it is not a limiting factor for using this apparatus, but it is desirable to plan—as a future perspective—a more controlled molding process. By reducing the variability of the sensitive elements we can aim at a more accurate measurement of the pressure spots.

With respect to the adopted pressure-sensitive technology a critical feature to discuss—and which could affect the system usability—is its expected lifetime under nominal (mechanical and electrical) operating conditions. For the estimation of the lifetime we did not carry out specific experimental tests. Rather, we carried out the following analysis, which led to the conclusion that the expected lifetime of the proposed sensorized mat is mostly affected by the life of off-the-shelf commercial components (*i.e.*, LED, ambient-light receivers, resistors) and is therefore sufficiently high to allow the use of the sensorized mat in prolonged clinical recordings.

The lifetime of the pressure-sensitive transduction units is determined by the life of the custom printed circuit board (PCB), the commercial electronic components soldered onto the PCB, and the silicone cover that is glued onto the PCB which deforms when loaded. The lifetime of the custom PCB can be affected by mechanical, electrical and/or thermal (temperature and humidity) stress. In order to mitigate the risk of failure from mechanical stress the PCB was built with a relatively high thickness—which enhances strength—and was housed into a rigid plastic frame that prevents it from bending. The use of voltage regulators and Zener diodes onto the supply lines protects the PCB (and electronic components) from voltage/current peaks, and consequent overheating. According to the current manufacturing procedures, complying with the industry standards set by the Association Connecting Electronics Industries (IPC^®^) [54]—under nominal working conditions (*i.e.*, 23 °C and about 30% relative humidity)—the lifetime of a 1.6-mm-thick PCB is guaranteed more than 10 years. The performance of the employed commercial electronic components gradually degrades over time. The degradation time is temperature and current dependant (the higher the working temperature and current, the shorter the lifetime of the component). Light emitters/receivers usually have a lifetime which is longer than 10 years under nominal working conditions. For instance, from [46,47,55] we know that the lifetime of the light transmitter is longer than 100,000 h (more than 11 years in continuous working mode) when it works at 84 °C and is supplied with a relatively high current (*i.e.*, 70 mA). These working conditions are much more stressful than the ones at which both light transmitters and receivers actually work in the sensorized mat (*i.e.*, room temperature: 25 °C, maximum supplied current: 1 mA). Finally, the lifetime of the silicone cover is mostly affected by the reliability of the glue and the mechanical strength of the silicone bulk as a consequence of either an overload or the application of cyclical load (loading-unloading cycles). With respect to the reliability of the glue (Sil-Poxy^®^, Smooth-On Inc., Easton, PA, USA; tensile strength: 5.17 MPa, breaking elongation: 750% [56]) we know from our many years of experience that the adopted solution ensures high-reliability. In the case of the sensorized insoles that we developed by exploiting the same pressure-sensitive technology and used to record more than 50,000 steps (*i.e.*, loading/unloading steps) in several experiments [20,57–60], we never reported any failure deriving from the ungluing of the silicone rubber cover from the PCB. For the sensorized mat we can expect a similar (or higher) reliability. Although the total glued surface is slightly smaller (because of the different size of the pyramidal frustum), the applied vertical and shear stress on the mat silicone-rubber cover is one order of magnitude smaller than the one applied on the insoles. Finally, with respect to the possible failure deriving from mechanical load, the following analysis applies. We used Dragon Skin^®^ Silicone which is a high-performance platinum-cure silicone featuring high strength and elasticity [61,62]. The minimum breaking stress (data extracted from the experimental characterization carried out by the Axel Products Inc., see Section 2) is about 0.17 MPa under traction and about 225 MPa under volumetric compression. In the case of the sensorized mat, the silicone cover is expected to be cyclically loaded with a maximum compressing pressure onto the top face of the pyramidal frustum of about 30 kPa. By means of the 3D FE model, we verified that this load should lead to a peak compression stress of 47 kPa and traction stress of 36 kPa, which are both much smaller (*i.e.*, 0.021% and 21.2% respectively) than the minimum breaking loads. As a consequence, under nominal loading conditions, the silicone rubber cover cannot break, and will have a lifetime which is at least comparable to the lifetime of the electronic components and PCB [63].

Usability of the system was finally proved by the experimentation with healthy infants. Results primarily showed that the system does not actually require any task specific calibration and it is easily portable into a clinical scenario. For all infants we could record consistent pressure maps at a sampling frequency of 10 Hz, which is sufficient to observe changes of infants' posture in prolonged rehabilitation sessions. The system was easily used by the experimenter, who was only requested to power on the system and switch on the data log at his preferred point in time.

The analysis of recorded pressure maps reveals that the system can localize several body parts over the three different tested postures. Over the five subjects, in addition to the pressure spots of the head, trunk/chest and pelvis, it was also possible to recognize spots corresponding to feet and hands. When looking at the pressure maps of the prone posture, the largest spot is the one of the chest, with a peak pressure between 15 kPa and 20 kPa (Subject #1). In three out of five subjects it was possible to recognize the location of hands and feet. In the case of the supine posture, in all subjects there is both a small spot (5–6 pixels) for the head and a larger spot for the trunk and pelvis, the latter spot being quite different among subjects and affected by the inter-subject anthropomorphic and postural variability. For example, Subject #5—who had a relatively small body weight and laid down on her left side—had a much smaller spot for the chest and pelvis than the one of Subject #4. An analogous analysis can be carried out for the seated posture. In Subjects #1 to #4 it is evident that the presence of spots for the pelvis and for the legs/feet (either grouped together or separate) are larger than the spots for Subject #5.

While being suitable to record consistent pressure maps in terms of spatial localization of pressure spots, further improvements are required to enhance accuracy in terms of pressure values of collected spots. Data in Table 4 shows that the mean recorded values of *vRF* (*i.e.*, the spatial integration of the pressure maps) either over- or underestimate the body weight. In particular, in the case of the prone posture, in all but Subject #2, *vRF* is lower than the bodyweight, with an error ranging between 12%–62%, while *vRF* for Subject #2 overestimates the body weight by 17.8%. For the supine posture, *vRF* underestimates the body weight (error in the range 15%–32%) for Subjects #1, #3 and #5, and overestimates (error in the range 10%–15%) for Subject #2 and #4. Finally, for the seated posture, *vRF* is higher than the body weight in all but Subject #5 (error in the range 6%–45%); underestimation for Subject #5 is about 50%.

The lack of a unique trend over all subjects and postures can be explained by two issues. On the one hand, there is the error introduced by the adopted force-to-output voltage numerical model (which can either overestimate or underestimate the actual force applied on each sensitive element). On the other hand, there is the fact that distribution of the load on the sensitive elements can be non-homogeneous (*i.e.*, the load on the top face of the pyramidal frustum is not equally distributed) and can result in either higher or smaller deformation of the silicone bulk compared to the nominal loading conditions, and—respectively—cause either overestimation or underestimation of the applied force.

It is worth noting that the recorded mean values of *vRF* have a standard deviation (expressed in percentage of the mean value) in the range of 5%–18%, 6%–13% and 3%–24%, respectively for prone, supine and seated postures. Actually, *vRF* variability results from the fact that the subjects were never fully still. In particular, reaction forces deriving from limbs, head and trunk movement were recorded by the sensorized mat.

Despite the limited accuracy in the estimation of the applied interaction force between the infant and the mat, this is not a limiting factor for using this apparatus to collect pressure maps, which bring exhaustive information about the spatial localization of pressure spots. We envision—as a future perspective—to improve the technology (*i.e.*, increase the system accuracy) mostly by increasing the spatial resolution. A higher spatial resolution—which means an area reduction of the top-face surface of the silicon cover of each sensitive element—will allow for a more homogeneous (and more similar to the nominal conditions) distribution of the applied load, and—as a consequence—improve accuracy.

## 5. Conclusions

In this paper we introduced a novel sensorized mat along with its experimental characterization. The device has a modular structure (with a minimum of one and a maximum of six sub-modules, and a total surface area of about 1 m^2^), and on-board electronics for data collection, pre-elaboration, and transmission to a remote computing unit for analysis and posture classification. On the one hand, results from the experimental characterization proved the usability of the system in a clinical setting and a prototypical application scenario. On the other hand, it is worth mentioning that in this manuscript we also advanced the knowledge about our pressure-sensitive technology (namely PSP2.1) by addressing the experimental characterization of 12 different sensitive elements (selected from six different silicone covers, molded separately). This allowed us to assess the variability of the mechanical properties derived from the hand-made molding procedure we used to develop the sensorized mat. Collected results also suggested—as a future objective—to improve system accuracy by means of a more controlled fabrication process and a higher spatial resolution. Finally, future work will also aim at developing automatic algorithms to monitor infants' movement from the collected pressure maps which will enhance the use of the developed system into multi-subject clinical trials with preterm infants.

## Figures and Tables

**Table 1.** Fitted model of the force-to-output voltage curve.

	**Fitting Model Coefficients (with 95% Confidence Interval)**				**Fit Goodness**		
**Sensor #**	***A*_1_**	***c*_1_**	***A*_2_**	***c*_2_**	**RMSE [N]**	**R^2^**	**RMSE [% fsr]**
**#1**	2.144 (1.885, 2.404)	1.651 (1.041, 2.261)	−2.382 (−2.661, −2.104)	−63.090 (−83.200, −42.970)	0.123	0.991	3.505
**#2**	2.328 (2.140, 2.516)	1.309 (0.937, 1.681)	−2.283 (−2.480, −2.087)	−56.430 (−68.020, −44.840)	0.086	0.995	2.462
**#3**	2.123 (1.904, 2.343)	0.9219 (0.614, 1.230)	−2.242 (−2.522, −1.963)	−55.290 (−74.14, −36.45)	0.135	0.988	3.983
**#4**	2.228 (1.996, 2.460)	1.303 (0.872, 1.735)	−2.282 (−2.533, −2.032)	−46.590 (−59.140, −34.040)	0.113	0.992	3.222
**#5**	1.892 (1.716, 2.069)	1.328 (0.986, 1.671)	−1.894 (−2.093, −1.695)	−58.890 (−75.800, −41.970)	0.101	0.993	2.900
**#6**	2.315 (1.954, 2.676)	1.342 (0.664, 2.020)	−2.399 (−2.800, −1.998)	−69.010 (−101.300, −36.700)	0.195	0.980	5.579
**#7**	2.006 (1.773, 2.239)	1.539 (1.158, 1.920)	−2.001 (−2.295, 1.706)	−61.090 (−87.970, −34.220)	0.155	0.988	4.424
**#8**	2.488 (2.166, 2.811)	1.153 (0.598, 1.708)	−2.452 (−2.775, −2.129)	−39.820 (−51.260, −28.370)	0.120	0.992	3.404
**#9**	2.053 (1.892, 2.213)	1.159 (0.881, 1.436)	−2.087 (−2.250, −1.924)	−33.920 (−40.500, −27.330)	0.067	0.996	1.915
**#10**	2.188 (1.717, 2.658)	1.903 (0.629, 3.178)	−2.153 (−2.630, −1.675)	−61.170 (−95.270, −27.070)	0.199	0.976	5.698
**#11**	2.287 (1.846, 2.727)	1.593 (0.473, 2.713)	−2.202 (−2.646, −1.759)	−62.980 (−92.800, −33.170)	0.177	0.981	5.055
**#12**	2.555 (2.194, 2.916)	1.164 (0.527, 1.802)	−2.545 (−2.897, −2.192)	−36.570 (−47.970, −25.160)	0.124	0.992	3.543
**All**	2.352 (2.235, 2.468)	0.999 (0.8051, 1.193)	−2.326 (−2.449, −2.203)	−44.840 (−50.820, −38.850)	0.221	0.962	6.303

**Table 2.** Hysteresis in percentage of the full-scale range of the sensitive elements.

**Sensor #**	**#1**	**#2**	**#3**	**#4**	**#5**	**#6**	**#7**	**#8**	**#9**	**#10**	**#11**	**#12**
**[% fsr]**	21.51%	11.72%	9.41%	10.10%	10.33%	8.26%	9.29%	14.62%	11.13%	15.94%	17.01%	10.77%

**Table 3.** Infants' age and weight.

**Subject data**	**Subject #1**	**Subject #2**	**Subject #3**	**Subject #4**	**Subject #5**
**Age [week]**	22	11	10	17	18
**Weight [kg]**	7.7	6.1	5.55	7.3	5.9

**Table 4.** Vertical reaction force (*vRF*): for each subject and posture the average value and standard deviation of *vRF* (μ ± σ) along with the percentage of variation with respect to the body weight are reported.

**Position**	**Prone**		**Supine**		**Seated**	

**Subject**	***vRF*[N]**	**Variation** **[%]**	***vRF*** **[N]**	**Variation** **[%]**	***vRF*** **[N]**	**Variation** **[%]**

**#1**	28.76 ± 4.14	−61.93	63.63 ± 3.84	−15.77	81.39 ± 4.56	+7.74
**#2**	70.50 ± 3.56	−17.81	68.84 ± 9.22	−15.04	77.69 ± 3.40	−29.83
**#3**	47.81 ± 2.84	−12.19	42.83 ± 4.42	−21.34	79.36 ± 2.86	+45.75
**#4**	37.45 ± 6.97	−47.70	78.71 ± 8.55	+9.91	75.63 ± 18.35	+5.61
**#5**	45.45 ± 3.74	−21.48	39.07 ± 5.45	−32.50	28.71 ± 1.69	−50.40
